# Unmet need for family planning and associated factors among women living with HIV in Gondar city, Northwest Ethiopia: cross-sectional study

**DOI:** 10.11604/pamj.2021.38.22.21431

**Published:** 2021-01-12

**Authors:** Mihret Dejen Kassie, Yohannes Ayanaw Habitu, Simegnew Handebo Berassa

**Affiliations:** 1 Teda Health Science College, Gondar, Ethiopia,; 2 Department of Reproductive Health, Institute of Public Health, College of Medicine and Health Sciences, University of Gondar, Gondar, Ethiopia,; 3 Department of Health Education and Behavioral Sciences, Institute of Public Health, College of Medicine and Health Sciences, University of Gondar, Gondar, Ethiopia

**Keywords:** Unmet need, HIV-positive women, ART, Ethiopia

## Abstract

**Introduction:**

all women, including those living with HIV, have the right to choose the timing, spacing, and number of their births and need access to family planning services. This study aimed at assessing the prevalence and factors associated with an unmet need for family planning among women receiving Antiretroviral Therapy (ART) services.

**Methods:**

a facility-based cross-sectional study was conducted from March to April 2018 in Gondar city, Ethiopia. A systematic random sampling technique was used to recruit 441 reproductive-age women on ART. The data were collected using a pretested structured questionnaire. The bivariate and backward multivariable logistic regression model was fitted to identify factors associated with the unmet need for family planning.

**Results:**

the prevalence of the unmet need for family planning among women living with HIV was 24.5%. Increase in women´s age (AOR: 0.90, 95% CI (0.85, 0.95)), having more than three children (AOR: 0.13, 95% CI (0.04, 0.38)), intention to have more children (AOR: 0.09, 95% CI (0.03, 0.23)), not disclosing sero-status to partner (AOR: 0.40, 95% CI (0.20, 0.82)) and having no experience of contraception use (AOR: 0.43, 95% CI (0.21, 0.90)) were protective factors against unmet need for family planning. Rural residence (AOR: 2.17, 95% CI (1.05, 4.46)) was associated with increased odds of unmet need for family planning.

**Conclusion:**

one in every four women living with HIV had an unmet need for family planning. So, continuous awareness-raising activities on family planning for women on ART should be given by emphasizing the rural and younger age women.

## Introduction

Globally, about 36.7 million persons are living with HIV, including estimated 1.8 million new infections in 2016. Of which 51% (17.8 million) are women aged 15 and older. Sub-Saharan Africa remains the most seriously affected region in the world [1]. Similarly, in Ethiopia an estimated 722,248 persons are living with HIV, women of reproductive age account for nearly 57% percent of all cases [2].

Family planning plays a vital part in preventing the transmission of HIV. It is the more cost-effective intervention for preventing mother to child transmissions (PMTCT) of the Human Immunodeficiency Virus (HIV), which is by far the main cause of HIV infection in children below the age of 15 years. In addition, family planning also prevents maternal morbidity and mortality that is caused by unintended pregnancy [3-8]. Unmet need for family planning is a robust indicator of the contraceptive utilization gaps [9]. Unmet need is the percentage of women who are married or in unions, fecund and sexually active, who wants to stop childbearing or delay their next birth by at least two years, but are not using any method of contraception, either modern or traditional [10-12].

The 2016 Ethiopian Demographic and Health Survey (EDHS) reported 58% of married women age 15-49 have a demand for family planning; 35% want to space births and 24% want to limit births. However, 22% of them have an unmet need for family planning: 13% to space or 9% limit births but are not currently using contraception. This unmet need for family planning in the country declined nearly by half over time, from 37% in 2000 to 22% in 2016 [12].

Pieces of evidence have shown that women who are living with HIV have a lower fertility desire and better use of contraceptives as compared to their HIV negative counterparts [13, 14]. Preventing unintended pregnancies among women living with HIV is one of the four comprehensive approaches that the World Health Organization (WHO) promotes to prevent the transmission of HIV from mother to baby [15].

Current evidences indicate that the unmet need for family planning among women living with HIV remains high in sub-Saharan Africa. Studies done in different African countries showed that unmet need for family planning was a range from Malawi 21.9% to 49% in Nigeria [16-22]. Regarding Ethiopia studies done in a different part of the country showed that the unmet need for family planning among HIV-positive women on ART ranges 15.4% in Nekemte, to 24.6% in South Gondar and North Wollo [23-25].

Studies conducted across multiple international contexts confirm the unique nature of FP's needs for HIV-positive women. This nature of the unmet need for family planning among HIV-positive women are different for each individual woman. Besides, the factors associated with this unmet need also vary across different contexts in the world. So, this study was conducted to determine the prevalence and associated factors of unmet need for family planning among HIV-positive women in Gondar city, Northwest Ethiopia.

## Methods

**Study design and setting:** a facility-based cross-sectional study was conducted from March to April 2018 at Gondar city, which is located at 727 km away from Addis Ababa. Gondar city is one of the administrative cities in the Amhara Region. One specialized hospital and eight health centers are providing health services in the city. There are 9,122 ART users in the city. (Gondar city health bureau, 2016 annual report, unpublished).

**Study participant and sampling:** all of reproductive age (15-49 years) women living with HIV and started ART, and had a follow up at ART clinics during the data collection period were included in the study. The sample size was determined by using the estimation of single population proportion formula with an assumption of 95% confidence interval, 4% margin of error, and 24.6% of an expected proportion of unmet need for family planning [25]. After considering the correction formula and compensation for the non-response rate, the final sample size was 441. In the city, five health centers were providing ART services. The total sample size was proportionally allocated to each health center based on the number of reproductive-age women who were receiving ART on each health center. A systematic random sampling method was employed to select participants in each of the facilities by using client flow as a sampling frame.

**Data collection tools and procedures:** a structured and pretested interviewer-administered questionnaire composed of socio-demographic characteristics, clinical, reproductive, communication and health facility factors were used for collecting the data. First, the questionnaire was prepared in English and translated into Amharic (the local language), and then translated back to English to check the consistency. Five nurses who have a Bachelor of Science in nursing, who are working in ART clinics, data collectors and two health officer supervisors were involved in the data collection process. On-site training was given for data collectors and supervisors on the objective of the research, on the procedure of data collection and ethical issues. The principal investigator and supervisor made day-to-day supervision during the data collection.

### Operational definition

**Unmet need for family planning:** unmet need for family planning was defined as the proportion of women with HIV who (1) are not pregnant and not postpartum amenorrhoeic and are considered fecund and want to postpone their next birth for 2 or more years or stop childbearing altogether but are not using a contraceptive method, or (2) have a mistimed or unwanted current pregnancy, or (3) are postpartum amenorrhoeic and their last birth in the last 2 years was mistimed or unwanted [12, 26].

**Data processing and analysis:** the collected data were entered, edited and cleaned using Epi info version 7 and analyzed using SPSS version 20. After the bivariable analysis was done, variables with p-values of < 0.2 were entered into a multivariable logistic regression model to identify factors associated with the unmet need for family planning. Adjusted odds ratios (AOR) with 95% confidence intervals (CI) were computed and P-value < 0.05 were considered statistically significant.

**Ethical consideration:** ethical clearance was obtained from the Institutional Review Board of the University of Gondar Institute of Public Health. A letter of permission to conduct the study was obtained from the Gondar city health office and each health center administrator. Informed consent was obtained from each study participant. To ensure confidentiality, names of the study participants were not recorded on the questionnaire and privacy was maintained during the interview. In addition, the collected data was kept locked in the file cabinet.

**Funding:** the authors received no specific funding for this work.

## Results

**Socio demographic characteristics:** a total of 441 reproductive-age women attending ART services were interviewed, with a response rate of 100%. The mean age of the participants was 33.59 + 5.31 years. More than half of them, 256 (58%) were urban dwellers. The majority of them were orthodox Christians 404 (91.6%) and 430(97.5%) Amhara. Two hundred forty-seven (56%) of the respondents were married. With regard to educational status, 139 (31.5%) were illiterate. One hundred forty-four (32.7%) of the women were housewives (Table 1).

**Table 1:** socio-demographic characteristics of HIV positive women on ART at Gondar city public ART clinics, Northwest Ethiopia, 2018

Variable		Frequency	Percent
**Residence**	Urban	257	58.3
	Rural	184	41.7
**Age**	19-24	23	5.2
	25-29	72	16.3
	30-34	125	28.3
	35-39	162	36.7
	Above 40	59	13.4
**Marital status**	Married	247	56.0
	Single	27	6.1
	Divorced	115	26.1
	Widowed	48	10.9
	Separated	4	0.9
**Religion**	Orthodox	404	91.6
	Muslim	34	7.7
	Catholic	3	.7
**Ethnicity**	Amhara	430	97.5
	Oromo	2	0.5
	Tigray	2	0.5
	Kimant	7	1.6
**Educational status**	Illiterate	139	31.5
	Able to read and write	62	14.1
	Completed Primary Education	115	26.1
	Completed Secondary Education	77	17.5
	College Diploma and above	48	10.9
**Occupational status**	Merchant	95	21.5
	Government employ	64	14.5
	Housewife	144	32.7
	Daily laborer	127	28.8
	Others	11	2.5
	Farmer	7	1.6
**Income**	Less than 850 ETB	110	24.9
	850-1005 ETB	111	25.2
	1006-2158.5 ETB	110	24.9
	Above 2159 ETB	110	24.9

**Communication characteristics of study participant:** of the participants, 336 (76.2%) knew the sero-status of their partner and of whom 87 (25.9%) were sero-positive. One hundred eighty-four (41.7%) of the respondents reported their sero-status for their partners. Two hundred eighty-five (64.6%) of the participant believed that contraceptive use might exacerbate HIV disease progression. Of all participants, 286 (72.8%) of them were supported by their partner in family planning service utilization.

**Reproductive and sexual characteristics:** three hundred twelve (70.7%) had less than three children and 238 (54%) had a desire for more children. Additionally, 302 (68.5%) of women had a history of sexual intercourse in the last six months and of whom 260(59%) had single sexual partner. Of the study participants, 299 (67.8%) of them had information on dual family planning methods and only 46 (19.1%) of them consistently used condom. Currently 108 (24.5%) of respondents did not use any contraceptive methods. Three hundred forty (77.1%) had ever used modern contraceptives. The most common modern family planning method used was injectable 206 (61.9%).

**Figure 1:**
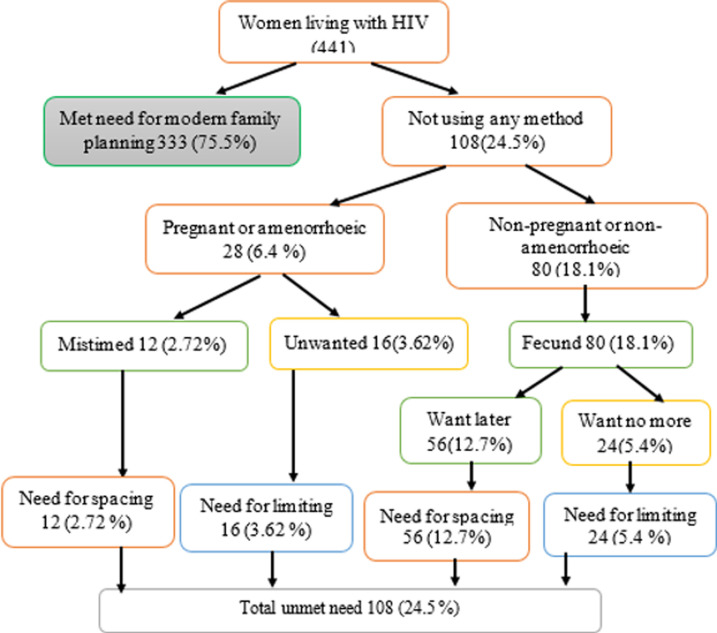
unmet need for family planning among HIV positive women on ART at Gondar city public ART clinics, Northwest Ethiopia, 2018

**Prevalence of unmet need for family planning:** the prevalence of unmet need for family planning was 24.5%, (95% CI: 20.4-28.8) of which 15.4% for spacing and 9.1% for limiting. Of the participants, 28 (6.4%) had an unplanned pregnancy and 80 (18.1%) were fecund, but were not pregnant and wanted to wait for two or more years (Figure 1**)**.

**Factors associated with unmet need for family planning:** in the multivariable logistic regression analysis, residence, age of the women, number of alive children, intention to have more children and ever use of contraception had a statistically significant association with unmet need for family planning. Women who live in rural places had 2.17 times higher odds of unmet need for family planning than those who live in urban (AOR: 2.17, 95% CI (1.05, 4.46)). The odds of unmet need for family planning decreased with the age of the women. One-year increase in the age of the women, the likelihood of having the unmet need for family planning decreased by 10% (AOR=0.90, 95% CI (0.85, 0.95)). The odds of unmet need for family planning among women who have more than three children was 87% less than those who have three and fewer children (AOR: 0.13, 95% CI (0.04, 0.38)). The odds of unmet need for family planning among women who do not intend to have more children was 91% less than those who intend to have more children (AOR: 0.09, 95% CI (0.03, 0.23)). The odds of unmet need for family planning among women who didn't disclose their sero-status to their partner was 61 % less than their counterparts (AOR: 0.40, 95% CI (0.20, 0.82)). The odds of unmet need for family planning among women who didn't ever used contraception was 57 % less than those who ever used contraception (AOR: 0.43, 95% CI (0.21, 0.90)) (Table 2).

**Table 2:** multivariable logistic regression of factors associated with unmet need for family planning among reproductive age women on ART at Gondar city public ART clinics, Northwest Ethiopia, 2018

Variables		Unmet need for family planning		COR (95% CI)	AOR (95% CI)
		yes	No		
Age in years (Mean (SD))		30.66 (5.01)	34.5(5.05)	0.86 (0.83,.90) **	**0.90 (0.85, 0.95)** **
Residence	Urban	62	195	1	1
	Rural	46	138	1.05(0.68, 1.63)	2.17(1.05, 4.46) *
Women educational status	Illiterate	22	117	1	1
	Able to read and write	12	50	1.28 (0.59, 2.78)	0.78(0.29, 2.05)
	Primary education	35	80	2.33 (1.27, 4.26) **	1.18(0.54, 2.57)
	Secondary education	25	52	2.56 (1.32, 4.94) **	0.78(0.33, 1.88)
	Above secondary	14	34	2.19(1.01, 4.74) *	0.64 (0.21, 1.95)
Husbands educational status	Illiterate	8	65	1	1
	Able to read and write	22	94	1.90 (0.80, 4.53)	1.20(0.42, 3.44)
	Primary education	35	62	4.5 (1.97, 10.66) **	2.31(0.83, 6.45)
	Secondary education	25	67	3.03(1.27, 7.21) *	1.22(0.42, 3.60)
	Above secondary	18	45	3.25 (1.30, 8.12) *	1.47(0.40, 5.46)
Husband occupation	Merchant	23	65	1	1
	Government employ	43	89	1.37 (0.75, 2.49)	1.13(0.50, 2.56)
	Daily laborer	16	37	1.22(0.57, 2.60)	1.05(0.41, 2.70)
	Private employ	23	124	0.52(0.27, 1.01)	0.50(0.22, 1.12)
	Farmer	3	18	0.47 (0.13, 1.75)	0.63(0.12, 3.26)
Number of alive children	Less than three	104	208	1	1
	Above three	4	125	0.064(0.023, 0.18) **	0.13 (0.04,0.38) **
Do you intend more children?	Yes	103	221	1	1
	No	5	112	0.10 (0.04-0.24) **	0.09(0.03, 0.23) **
Do you disclose your status to your partner?	Yes	54	130	1	1
	No	54	203	0.64(0.414-0.99) *	0.40(0.20, 0.82) *
Do you ever use condom?	Yes	68	173	1	1
	No	40	160	0.64(0.41-0.99) *	0.76(0.43,1.36)
Do you ever use contraceptive?	Yes	92	248	1	1
	No	16	85	0.51(0.28-0.91) *	0.43(0.21, 0.90) *
Do you ever acquire STI?	Yes	19	43	1	1
	No	89	290	0.70(0.39-1.25)	0.67(0.31, 1.41)
Duration on ART	<6 months	70	166	1	1
	>6 months	38	167	0.54(0.34-0.85) *	0.92(0.52, 1.62)

* p-value < 0.05 **p-value <0.01

## Discussion

This study aimed to measure the magnitude of the unmet need for family planning and its associated factors among HIV-positive women on ART. According to this study´s finding, the prevalence of unmet need for family planning was 24.5% (95% CI: 20.4-28.8). This finding was in line with studies done in, North Tanzania, Ghana, South Gondar and North Wollo [17, 18, 25]. This may be due to the similarity of participants´ characteristics and the Ethiopian government is striving to address unmet need for family planning.

However, the finding was lower than the studies done in Nsambya (45.1%) and Mulago at 30.9% clinics in Uganda, Kampala, Nairobi Kenya 33.6% and Nigeria 49% [19, 20, 22]. This discrepancy might be due to the difference in health service provision, initiation and scaling up of health extension workers and consistent implication of sustainable development goals, investment focus on maternal health by the government and awareness creation towards health through health development army were some inputs for the lower record of our findings [5]. This finding was higher than the findings in Nekemt 15.4% and Hawasa 19.1% [23, 24]. The possible reasons may be due to behavioral differences, the difference in health service access and utilization.

In this study, the residence of HIV-positive women was significantly associated with the unmet need for family planning. Rural women on ART have almost 2 times more experience of unmet need for family planning as compared to those who live in urban. This result is supported by Ethiopian health and demography survey finding for the general population [12]. This is due to difficulties to the difference in the distribution of health facilities, and access to roads and public transport [27, 28].

The age of the women was negatively associated with the unmet need for family planning. As the age of the women increased their unmet need for family planning decreases. This finding is similar to a study done in Hawassa, South Ethiopia [24]. In addition, this finding is similar to the survey results in the general population in Ethiopia; in which the unmet need for family planning is significantly higher among women of younger age groups as compared with old aged women [29]. This might be due to raised awareness of family planning as a result of expanded school-based family planning programs. This finding contradicted with a study done in Nsambya district, Uganda; where older women more likely have the unmet need for family planning [20]. Lower perception of the risk of pregnancy, and access with youth-friendly services, school health, and integrated services result in such differences [29, 30]. Women who already had more than three children have 88% lower risk to unmet need for family planning than those who have three and fewer children. This mean the more the number of alive children the woman had, the lower the chance of having unmet need for family planning methods. Similarly, in this study women who do not have an intention to have more children have a 92% lower unmet need for family planning. This finding was inconsistent with studies done in Kenya, Malawi and Nigeria [19, 20, 22]. Multiparous women will have more need of contraception to limit the number of children they have compared to women with no or few children. The difference may be due to a lower perception of the risk of pregnancy as the women aged, have no intention to be pregnant and had more children. In this study, women who do not disclose their HIV sero-status for their partner have a 61% lower risk of the unmet need for family planning than those who did. This finding specifies disclosure of their status to their partner is a key to making informed decisions positive health outcomes. All women should be encouraged to do so as much as possible [18].

This study finding also revealed that women who previously had no experience of contraceptive use were 56% less likely to have the unmet need for family planning. This study finding differs from study done in Nigeria [22]. As women had previous experience of using a method were more likely need to use it. But, due to health service accessibility issues, they may have an unmet need for family planning.

### Strengths and weaknesses of the study

The findings were based on the data collected from the health center, the primary health care level. This study has some limitations. The primary limitation of the study was the use of cross-sectional data, which rule out the analysis of causal association. On the other there was a risk of social desirability bias whereby women living with HIV may over-report their contraceptive use (condom use). The questionnaire was administered by nurses who were working in ART clinics to minimize this bias.

## Conclusion

The prevalence of unmet need for family planning among HIV-positive women was high. Place of residence, age of the women, number of alive children, intention to have more children, and ever use of contraception were the main factors associated with the unmet need for family planning. So, continuous awareness-raising activities on family planning for women on ART services is required. In addition, professionals should give due emphasis to rural and younger age women.

### What is known about this topic

- Contraception utilization of HIV infected women;
- Family planning methods mostly used by HIV infected women;
- Unmet need for family planning among general women population and HIV positive women on ART.

### What this study adds

- The current prevalence of unmet need for family planning among HIV infected women at health centers, the primary health care level;
- The effect of communication factor like husband opposition, disclosing one´s sero-status, and number of partners on unmet need for family planning;
- Factors associated with unmet need for family planning among HIV infected women on ART at health centers.
